# In vitro biological evaluation of eight different essential oils against *Trypanosoma cruzi*, with emphasis on *Cinnamomum verum* essential oil

**DOI:** 10.1186/1472-6882-14-309

**Published:** 2014-08-22

**Authors:** Camila Maria O Azeredo, Thalita Gilda Santos, Beatriz Helena Lameiro de Noronha Sales Maia, Maurilio José Soares

**Affiliations:** Laboratory of Cell Biology, Carlos Chagas Institute/Fiocruz, Rua Prof. Algacyr Munhoz Mader 3775, Cidade Industrial, 81350-010 Curitiba, PR Brazil; Laboratory of Natural Products and Chemical Ecology, Department of Chemistry, Federal University of Paraná (UFPR), 81.531-990 Curitiba, PR Brazil

**Keywords:** Cinnamon, *Cinnamomum verum*, Essential oil, *Trypanosoma cruzi*

## Abstract

**Background:**

Essential oils (EOs) are complex mixtures of secondary metabolites from various plants. It has been shown that several EOs, or their constituents, have inhibitory activity against trypanosomatid protozoa. Thus, we analyzed the biological activity of different EOs on *Trypanosoma cruzi*, as well as their cytotoxicity on Vero cells.

**Methods:**

The following EOs were evaluated on *T. cruzi* epimastigote forms: *Cinnamomum verum*, *Citrus limon*, *Cymbopogon nardus*, *Corymbia citriodora*, *Eucalyptus globulus*, *Eugenia uniflora*, *Myrocarpus frondosus*, and *Rosmarinus officinalis.* Inhibitory activity against *T. cruzi* (IC_50_/24 h) and cytotoxicity against Vero cells (CC_50_/24 h) were evaluated by the MTT assay. The EO of *C. verum* was selected for further evaluation against trypomastigotes and intracellular amastigotes, as well as on parasite metacyclogenesis. Constituents of *C. verum* EO were identified by GC-MS. One-way ANOVA statistical analysis was performed with GraphPad version 5.01.

**Results:**

*Cinnamomum verum* EO was the most effective against *T. cruzi* epimastigotes (IC_50_/24 h = 24.13 μg/ml), followed by *Myrocarpus frondosus* (IC_50_/24 h = 60.87 μg/ml) and *Eugenia uniflora* (IC_50_/24 h = 70 μg/ml). The EOs of *C. citriodora*, *E. globulus*, and *R. officinalis* showed no activity at concentrations up to 300 μg/ml. Incubation of *T. cruzi* metacyclic trypomastigotes and intracellular amastigotes with *C. verum* EO resulted in IC_50_/24 h values of 5.05 μg/ml and 20 μg/ml, respectively. Therefore, trypomastigotes are more susceptible than epimastigotes, with selectivity index (SI) about 4.7-fold higher (9.78 and 2.05, respectively). Analysis of *C. verum* EO by GC–MS showed mainly *(E)*-cinnamaldehyde (81.52%) and eugenol (16.68%).

**Conclusions:**

*C. verum* essential oil is effective against *T. cruzi* (epimastigotes, trypomastigotes and amastigotes) and interferes with the parasite differentiation process in vitro. Thus, it represents a strong candidate for further studies to improve its activity on pathogenic trypanosomatids.

## Background

Chagas disease, caused by the flagellate protozoan *Trypanosoma cruzi*, is a chronic disease that occurs mainly in Latin America. It is estimated that 7–8 million people are infected worldwide [1]. Most infected people live in endemic areas, comprising 21 Latin America countries [1]. Chagas disease is technically considered a zoonosis, as the natural reservoirs are marsupials and placental mammals. The disease in humans results from the invasion of natural ecotopes and the establishment of vectors in human dwellings in endemic areas because of poor socio-economic conditions in most rural populations [2].

*T. cruzi* is naturally transmitted by blood-sucking insects of the subfamily Triatominae (Hemiptera: Reduviidae). Human infection occurs usually by insect bite, oral transmission, blood transfusion, or congenital transmission [2–4]. Transmission through blood transfusion, congenitally, and with intense international migration, has led to spread of the disease to non-endemic regions, such as United States and Western Europe [2].

Two drugs emerged in the late 1960s for the treatment of Chagas disease: benznidazole (Rochagan in Brazil and Radanil in Argentina, from Roche) and nifurtimox (Lampit, from Bayer). These two drugs are still the only ones available for Chagas disease. Both drugs were originally recorded for treatment of the acute phase of Chagas disease, but are currently used in both acute and initial chronic phases [2, 5]. However, these chemotherapeutic drugs do not completely fill the World Health Organization (WHO) criteria for an ideal drug, which are: (i) parasitological cure in acute and chronic cases of infection, (ii) effectiveness in a single dose or a few doses, (iii) accessibility to patients (low cost and easy to achieve), (iv) no teratogenic or side effects, (v) no need for hospitalization of patients for treatment, and (vi) without showing resistance or induction of resistance to the etiological agent [6]. Furthermore, efficacy in the acute phase varies with the geographical area, probably because of differences in susceptibility between different strains of *T. cruzi* [2].

Maintenance of new cases in endemic regions and the recent spread of the disease into non-endemic regions point towards the need to find drugs that are effective both in treatment of disease and prophylaxis of *T. cruzi* in blood banks. Various drugs for the treatment of parasitic diseases have been extracted from plants or synthesized from vegetal prototypes [7, 8]. Therefore, the study of extracts and compounds with biological activity isolated from plants used in folk medicine is promising in the search for compounds with potential for the prophylaxis of Chagas disease.

Essential oils (EOs) are complex mixtures of secondary metabolites isolated from various plants, which may be synthesized by all plant organs. In these mixtures there are 20–60 constituents at different concentrations, but usually only 2–3 major constituents determine the biological properties of the EO [9]. Essential oils and their constituents present a broad pharmacological spectrum, and are used as antimicrobials, analgesics, sedatives, anti-inflammatory, and anti-spasmodic drugs, as well as anthelmintics and antiprotozoals [6, 9–13].

It has been recently shown that several EOs, or their constituents, have inhibitory activity against trypanosomatid protozoa [6, 14–22]. However, most studies in *T. cruzi* evaluated the inhibitory activity only on culture epimastigotes and blood trypomastigotes. The data are sparse on the effect of EOs, or their main constituents, on cell differentiation (metacyclogenesis) and on *T. cruzi* intracellular amastigotes. Thus, we have analyzed the effect of different EOs, with emphasis on *Cinnamomum verum* EO, on epimastigotes, trypomastigotes, and amastigotes, as well as on the process of differentiation in vitro (metacyclogenesis) of *T. cruzi*.

## Methods

### Essential oils (EOs), (E)-cinnamaldehyde and Benznidazole

Essential oils of *Cinnamomum verum* (formerly *Cinnamomum zeylanicum;* Lauraceae; cinnamon) bark, *Citrus limon* (Rutaceae; lemon), *Cymbopogon nardus* (Poaceae; citronella grass), *Corymbia citriodora* (formerly *Eucalyptus citriodora*; Myrtaceae; lemon eucalyptus), *Eucalyptus globulus* (Myrtaceae; blue gum), *Eugenia uniflora* (Myrtaceae; pitanga), *Myrocarpus frondosus* (Fabaceae; cabreúva), and *Rosmarinus officinalis* (Lamiaceae; rosemary) were purchased from QUINARI Cosmetic and Fragrances Inc. (Maringá-PR, Brazil). EOs from cinnamon and lemon eucalyptus were from lot 05209; EOs of pitanga, blue gum, cabreúva, and lemon were from lot 022186; citronella grass EO was from lot 519/520; and rosemary EO was from lot 022185. All EOs were valid until August 2012 and used from 2011–2012. Benznidazole and *(E)-*cinnamaldehyde were purchased from Sigma-Aldrich (St Louis, MO, USA).

EOs, (*E*)-cinnamaldehyde and benznidazole were first diluted in dimethylsulfoxide (DMSO) at 100 mg/ml for EOs, 50 mg/ml for benznidazole (192.14 mM), and 13.216 mg/ml (100 mM) for (*E*)-cinnamaldehyde (first stock). For use, the first stock was diluted 1:50 (benznidazole) or 1:100 (EOs and cinnamaldehyde) in either LIT (liver infusion tryptose) or RPMI-1640 (Sigma-Aldrich) media (second stock). Therefore, DMSO was diluted to 1%, ensuring that its final concentration in the experiments never exceeded 0.5%, a concentration that is not harmful to parasites and Vero cells. These stocks were stored at 4°C in the dark, to avoid degradation [23]. The second stock was prepared minutes before use.

### Chemical composition of Cinnamomum verum essential oil

Gas chromatography–mass spectrometry (*GC–MS)* analysis was performed using a Shimadzu GC-2010 gas chromatograph coupled with GCMS-QP2010 Plus equipped with auto sampler (model AOC-20i, Shimadzu, Columbia, MD, USA) and GC–MS Solution software. Analysis was performed with a Rtx-5MS capillary column (30 mm × 0.25 mm × 0.25 μm), using temperature programmed condition from 60°C to 250°C at 3°C/min. Analysis conditions were: injector temperature 250°C, ion source interface temperature 300°C, analysis of masses between 40–350 *m/z*, electron impact at 70 eV, column head pressure at constant pressure of 59 kPa, column flow 1.02 ml/min, gas linear velocity: 36.8 cm/s, carrier gas: helium, injected volume: 1 μl (1% dilution in hexane) in split mode (ratio 1:10). Constituents of the *C. verum* essential oil were identified by comparing their mass spectral pattern and retention indices (RI) relative to a standard *n*-alkane series (C_9_–C_24_) with those given in the literature [24] and the Wiley 138 and Nist 98 databases.

### Evaluation of EO activity on T. cruzi epimastigotes

Epimastigotes (strain Dm28c) were maintained at 28°C in LIT medium supplemented with 10% fetal calf serum (FCS), with weekly passages. For the screening of EO activity, epimastigotes were collected from cultures at the mid log phase of growth (3-days-old). Parasite concentration was adjusted to 1 × 10^7^ cells/ml and 180 μl/well were added to a 96 well plate (1.8 × 10^6^ parasites/well). Then 20 μl of compounds (final concentration: 5–300 μg/ml for EOs, 0.26–39.65 μg/ml [2–300 μM] for *(E)-*cinnamaldehyde, and 6.25–150 μg/ml [0.024–0.576 mM] for benznidazole) were added to each well. In control wells, 20 μl of culture medium were added without the compounds. The plates were incubated for 24 h at 28°C and then 50 μl of MTT (at 10 mg/ml in PBS) was added to each well (final concentration: 2 mg/ml per well). The plates were wrapped in aluminum foil, incubated for 3 h at 37°C and then centrifuged at 475 *g* for 10 min. The supernatant was removed by abrupt plate inversion. Then 20 μl of 10% SDS in 0.01 M HCl was added and the parasites were resuspended by gently tapping the plates. The plates were then incubated at 37°C for 1 h. Thereafter, 80 μl of pure DMSO was added to all wells to solubilize the formazan crystals. Optical density (OD) was read at 550 nm in an ELISA reader (Biotek model ELx800; Biotek, Winooski, VT, USA). Mean of at least two independent experiments was used to calculate the IC_50_/24 h using Microsoft Excel software by linear correlation. Each experiment was performed in triplicate.

### Evaluation of EO activity on T. cruzi metacyclic trypomastigotes

Purified metacyclic trypomastigotes were obtained in vitro after nutritional stress in TAU3AAG medium, as previously described [25]. Briefly, 150 cm^2^ bottles were inoculated with 5 × 10^6^ epimastigotes/ml and after 72 h at 28°C metacyclic trypomastigotes were released into the supernatant. The cells were collected and purified by passage through an affinity column containing DEAE cellulose resin equilibrated with phosphate-saline-glucose buffer (PSG: 47.47 mM Na_2_HPO_4_, 2.5 mM NaH_2_PO_4_ · H2O, 36.76 mM NaCl, 55.5 mM glucose). After purification, the concentration of trypomastigotes was adjusted to 5 × 10^6^ cells/ml and they were distributed into 24-well plates containing TAU3AAG medium with different concentrations (0–20 μg/ml) of *C. verum* EO. Cell lysis was determined after 24 h by counting parasite density with a Neubauer chamber. This density was used to calculate the IC_50_/24 h (concentration leading to 50% cell lysis). The experiment was performed in triplicate.

### Evaluation of EO activity on T. cruzi amastigotes

Vero cells (ATCC: CCL-81) were kept at 37°C in a humidified 5% CO_2_ incubator, in 25 cm^2^ culture flasks containing RPMI-1640 medium pH 7.4 supplemented with 2.5% FCS, 2 mM L-glutamine, 10 μg/ml streptomycin and 10 μg/ml penicillin. For the experiments, the cells were seeded into 24-well plates and then infected with trypomastigotes at a 10 parasites/cell ratio. After 4 h the monolayers were washed with PBS to remove non-internalized parasites and kept for 12 h at 37°C in 1 ml RPMI/2.5% FCS in a 5% CO_2_ humidified atmosphere. After that, the EOs were added to the RPMI medium at different concentrations (5–20 μg/ml) and the plates were incubated for 24 h. The cells were then stained with Giemsa and the whole well (total of nine fields) was photographed in a Nikon TE300 inverted light microscope, with a 20× objective.

Amastigote counting was performed using ImageJ software, observing the percentage of infected cells, the number of amastigotes/cell, and the total number of amastigotes/well. Density of amastigote population was calculated dividing the total number of amastigotes/well by the well area (201 mm^2^). The percentage of inhibition (%I) was calculated according to Guru et al. (1989) [26], as modified by Lakshmi and collaborators (2007) [27], using the following formula: %I = 100 − (T/C × 100), where T is the total number of intracellular amastigotes in treated cells and C is the total number of intracellular amastigotes in control cells. The IC_50_/24 h value (concentration that inhibits proliferation of intracellular amastigotes by 50%) was estimated from the%I value with the Microsoft Excel software by linear correlation. Statistical analysis (one-way ANOVA) of the data was performed using the software GraphPad Prism Version 5.01 and data with p < 0.05 were considered significantly different. The experiment was performed in triplicate.

### Evaluation of EO activity on T. cruzi metacyclogenesis

Epimastigotes at late log phase of growth (cell density of 5–7 × 10^7^ cells/ml) were collected by centrifugation for 5 min at 7000 *g* at 10°C, resuspended in triatomine artificial urine (TAU) medium at 5.0 × 10^8^ cells/ml and kept at 28°C for 2 h. Then, corresponding with nutritional stress, the cells were transferred to 25 cm^2^ bottles containing 5 ml of TAU3AAG medium (final concentration of 5.0 × 10^6^ cells/ml) with different concentration of *C. verum* EO (4–145 μg/ml) and incubated at 28°C. After 24 h, the relative number of epimastigotes and trypomastigotes was counted with a Neubauer chamber and used to calculate the percentage of differentiation. Mean inhibition of differentiation for each concentration (as compared with metacyclogenesis in untreated control cultures) was used to calculate the IC_50_/24 h using Microsoft Excel software by linear correlation. The experiment was performed in triplicate.

### Cytotoxicity

Uninfected Vero cell monolayers were washed with PBS pH 7.2, detached by treatment with 0.25% trypsin/0.1% EDTA for 5 minutes at 37°C, washed with RPMI medium pH 7.4 + 2.5% FCS, centrifuged at 0.2 *g* for 10 min at 4°C and resuspended in the same medium. Cell viability was assessed by Trypan Blue staining and the cells were seeded into 96-well plates (2 × 10^4^ cells/well). After 24 h, the cells were incubated with EOs or cinnamaldehyde at different concentrations (25–1000 μg/ml for EOs; 0.26–39.64 μg/ml [2–300 μM] for cinnamaldehyde). After 24 h of incubation, integrity of the cell monolayer was observed under an inverted microscope and 50 μl of MTT (at 2 mg/ml in PBS) was added. After 4 h of incubation, absorbance was read at 550 nm with an ELx800 (BioTek) microplate reader. The mean of at least two independent experiments was used to plot a graph of inhibition × concentration, which was used to calculate the CC_50_ (50% cytotoxic concentration) using Microsoft Excel software by linear correlation. Each experiment was performed in triplicate.

## Results

An initial screening of all EOs was performed on *T. cruzi* epimastigotes, evaluating inhibitory activity at concentrations of 50 μg/ml and 300 μg/ml. In this first trial, the EOs of *Rosmarinus officinalis*, *Eucalyptus globulu*s, and *Corymbia citriodora* did not show activity at 300 μg/ml and were therefore not further evaluated.

Lower concentrations of the remaining EOs were then evaluated to estimate the IC_50_/24 h (Table 1). The most effective EO was that of *C. verum* (IC_50_/24.13 ± 1.13 μg/ml; IC_90_/24 h = 48.33 μg/ml), followed by *M. frondosus* (IC_50_/24 h = 60.87 ± 1.13 μg/ml), and *E. uniflora* (IC_50_/24 h = 70 ± 1.04 μg/ml). The IC_50_/24 h of the reference drug benznidazole was 15.8 ± 1.75 μg/ml (61 μM). Evaluation of cytotoxicity on Vero cells showed that the least cytotoxic EO was that of *C. limon* (CC_50_/24 h = 281.69 ± 1.12 μg/ml). EOs with higher selectivity indexes were those from *C. limon* (SI = 2.63), *E. uniflora* (2.46), *M. frondosus* (2.32), and *C. verum* (2.05), but all were more cytotoxic and less selective than benznidazole (CC_50_/24 h = 147.37 ± 1.22 μg/ml; SI = 9.33).

**Table 1:** **EO activity on** ***T. cruzi*** **(IC**_**50**_ **/24 h) epimastigotes and Vero cells (CC**_**50**_ **/24 h)**

Essential oil	IC _50_epimastigotes (μg/ml)	CC _50_Vero cells (μg/ml)	Selectivity index (SI)
*Cinnamomum verum*	24.13 ± 1.13	49.4 ± 1.12	2.05
*Myrocarpus frondosus*	60.87 ± 1.13	141.3 ± 1.14	2.32
*Cymbopogon nardus*	94 ± 1.14	178.95 ± 1.1	1.90
*Citrus limon*	107.14 ± 1.03	281.69 ± 1.12	2.63
*Eugenia uniflora*	70 ± 1.04	172.42 ± 1.15	2.46
Benznidazole (reference drug)	15.8 ± 1.75 (61 μM)	147.37 ± 1.22	9.33

Since the *C. verum* EO was notably more effective on *T. cruzi* epimastigotes, it was selected for further evaluation on other developmental forms (trypomastigotes and amastigotes), as well as on the parasite differentiation process (metacyclogenesis). Metacyclogenesis was not significantly affected by *C. verum* EO at concentrations up to 15 μg/ml. Higher concentrations led to reduction in percentage of trypomastigotes and metacyclogenesis was totally abolished with 25 μg/ml (IC_50_/24 h = 18.2 ± 1.04 μg/ml). After 48 h of treatment with the IC_50_/24 h value, the surviving cells showed slower motion compared with the control. After treatment for 24 h with 35 μg/ml (2 × IC_50_/24 h) the few remaining cells (epimastigote forms) were motionless.

On purified metacyclic trypomastigotes, the estimated IC_50_/24 h was 5.05 ± 1.03 μg/ml (IC_90_ = 8.21 μg/ml), showing that this form is more susceptible than epimastigotes. The selectivity index (SI) increased more than 4-fold as compared with epimastigotes (9.78 and 2.05, respectively).

**Figure 1:**
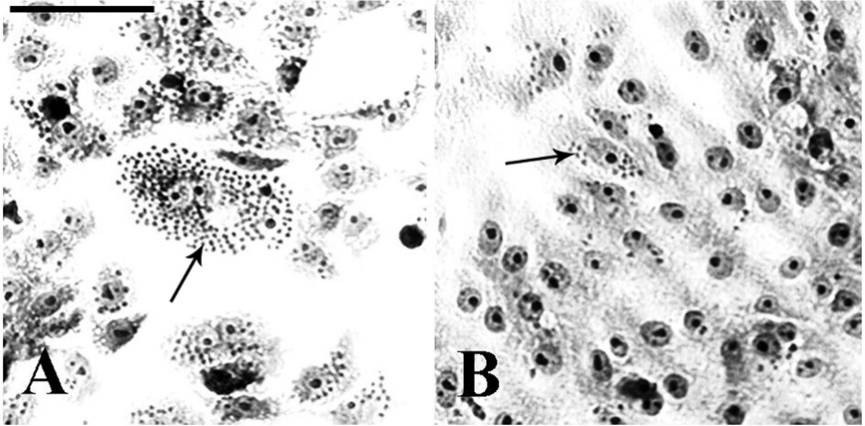
**Vero cells infected with** ***T. cruzi*** **and then treated with** ***C. verum*** **essential oil (EO). (A)** Control, without treatment; **(B)** Treatment for 24 h with 20 μg/ml *C. verum* EO. Note the decrease in amastigote (arrows) number after treatment. Bar = 100 μm.

**Figure 2:**
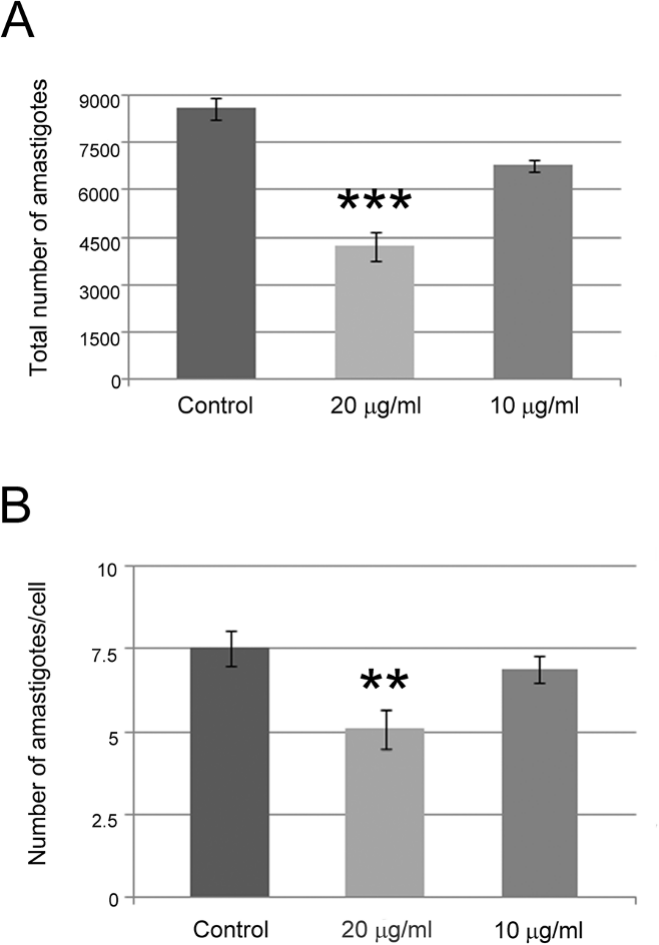
**Number of intracellular amastigotes in Vero cells infected with** ***T. cruzi*** **.** Amastigote counting was performed in cultures without treatment (control) or after treatment for 24 h with 10 or 20 μg/ml *C. verum* EO. **(A)** Number of total amastigotes per well; **(B)** Number of amastigotes per cell. ***Statistically different from control and treatment with 10 μg/ml (p < 0.0001); **Statistically different from control and treatment with 10 μg/ml (p < 0.0029).

Vero cells were first infected and then incubated with *C. verum* EO, to assure that the effect was on amastigote proliferation and not on adhesion/penetration of the trypomastigotes used in the infection. Treatment with 20 μg/ml reduced the number of total amastigotes by 50% when compared with control infection, resulting in an IC_50_/24 h value of 20 μg/ml (Table 2; Figures 1 and 2). The number of amastigotes/mm^2^, the association index, and the percent inhibition (%I) were also reduced by half, when compared with the untreated infection (Table 2). The mean number of amastigotes per cell decreased from 7.52 (untreated control) to 5.08 (Table 2; Figure 2).

**Table 2:** **Effect of** ***C. verum*** **EO on** ***T. cruzi*** **intracellular amastigotes after treatment for 24 h**

	% Infected cells	Total intracellular amastigotes	Amastigotes/cell (mean)	Density amastigotes/mm ^2^	AI ^a^	%I ^b^
Control	59 (n = 5812)	25746	7.52	42.7	442.5	-
10 μg/ml	51.8 (n = 5709)	20315	6.87	33.7	355.87	21.1
20 μg/ml	50.6 (n = 4946)	12717	5.08	21.1	257.05	50.61

^a^Association Index (AI) =% infected cells × amastigotes/cell.

^b^% Inhibition (%I) = 100 − (T/C × 100), where T is the total number of intracellular amastigotes in treated cells and C is the total number of intracellular amastigotes in control cells.

GC–MS analysis identified five main constituents in *C. verum* EO, the major constituents being *(E)-*cinnamaldehyde (81.52%) and eugenol (16.68%), followed by *(E)*-caryophyllene, (E)-cinnamyl acetate, and α-humulene (Table 3). Since *(E)*-cinnamaldehyde was identified as the main constituent, it was further evaluated against *T. cruzi* epimastigotes and Vero cells, by the MTT colorimetric assay. However, it showed no activity or cytotoxicity at low concentrations up to 39.65 μg/ml (300 μM).

**Table 3:** ***Cinnamomum verum*** **EO constituents by GC–MS analysis**

Constituent	Retention time	Concentration (%)	RI calc	RI lit
*(E)-*cinnamaldehyde	20.115	81.52	1282	1267
eugenol	23.713	16.68	1366	1356
*(E)-*caryophyllene	26.274	1.19	1426	1417
*(E)-*cinnamyl acetate	27.280	0.01	1450	1443
α-humulene	27.709	0.12	1460	1452

RI calc: Retention Index calculated; RI lit: Retention Index from the literature [24].

## Discussion

Interest is growing in the search for natural compounds active against pathogenic trypanosomatids, resulting in several reports on the biological activity of essential oils (EOs) or their main constituents on these protozoa [6, 18–20]. This activity is probably related to the function of EOs in nature, where they play a protective role in plants, acting as antibacterial, antiviral, antifungal, and protection against herbivory [9].

We have here first screened the activity of different EOs on *T. cruzi* culture epimastigotes, which are easier to grow and thus represent a simple model for the identification of potential compounds active against this parasite. However, in vitro activity against *T. cruzi* epimastigotes does not guarantee promising activity against other forms of the parasite. In fact, a considerable number of plant extracts with positive inhibitory effects in vitro do not become alternative chemotherapies [28]. Therefore, a large number of EOs or their constituents and derivatives should be analyzed, until a promising molecule acting on different developmental stages of *T. cruzi* can be obtained.

Among the eight essential oils that we analyzed, the *C. verum* EO showed the most activity against *T. cruzi* epimastigotes. It has been shown that *Cinnamomum* sp. EO has antipyretic, antibacterial, antifungal, antiparasitic, and repellent activities [9, 28–31]. However, despite its good inhibitory activity on *T. cruzi*, its IC_50_/24 h was still higher than that of the reference drug benznidazole, suggesting the need for high concentrations for in vivo studies. Nevertheless, it has been shown in treatment of mice that high concentrations of *Cymbopogon citratus* EO produce no toxic effects [32]. This finding indicates that EOs (or their main constituents) may have better activity in vivo than in vitro, with activity on parasitic infections and no cytotoxic activity [32, 33]. Furthermore, treatment of mice with EOs showed beneficial effects not related to the parasite infection, such as reductions in plasma cholesterol [32]. Therefore, *C. verum* EO (or cinnamaldehyde derivatives) is a potential candidate in the search for trypanocidal chemotherapeutic drugs.

Although it did not have the best selectivity index (SI) on epimastigotes, the *C. verum* EO was effective at a concentration much lower than that of the other EOs that we evaluated. It was also effective on *T. cruzi* epimastigotes at concentrations lower than those previously obtained with EOs from *Origanum vulgare* (oregano), *Thymus vulgaris* (thyme), *Achillea millefolium* (yarrow), *Syzygium aromaticum* (clove), *Ocimum basilicum* (basil), and *Cymbopogon citratus* (lemon grass), or their main constituents [18–20]. Activity of the *C. verum* EO was also higher than that obtained with other EOs used against other pathogenic trypanosomatids [14, 16, 17, 34].

On *T. cruzi* amastigotes, the *C. verum* EO was effective with IC_50_/24 h = 20 μg/ml (SI = 2.47), a value similar to that found with epimastigotes. Its activity was better than that obtained with jacaranone (main constituent of *Pentacalia desiderabilis*), which showed no activity on intracellular amastigotes of *T. cruzi* and *Leishmania chagasi* at a concentration of 100 μg/ml [35]. However, the IC_50_/24 h value found here was four times higher than that obtained by Santoro and colleagues [18] with *Cymbopogon citratus* EO (IC_50_/24 h = 5.1 μg/ml), although the SI was similar to that obtained with *Lippia alba* EO [36].

It has been shown that citral, the main constituent of *Cymbopogon citratus* EO, was able to inhibit differentiation of *T. cruzi* at a concentration of 30.8 μg/ml [37]. Our data showed that *C. verum* EO also interfered with metacyclogenesis of this parasite. The differentiation process was totally inhibited with 25 μg/ml. At this concentration, only epimastigotes forms could be observed in the culture supernatant. Although it is possible that the EO was killing the trypomastigote forms, we cannot exclude the possibility that the inhibitory effect could be also due to killing of epimastigotes prior to the differentiation process, thus lowering the number of resulting trypomastigotes.

The *C. verum* EO presented higher activity on *T. cruzi* purified metacyclic trypomastigotes, increasing the SI to 9.78. This increase in SI, as compared with epimastigotes, has been already observed [18, 19]. Such difference may be related to diverse metabolic pathways and membrane composition in the various developmental forms of *T. cruzi*. It is also possible that the composition of the different culture media used for the various forms of *T. cruzi* (LIT, RPMI-1640 and TAU3AAG media) may influence absorption and/or degradation of the EOs.

Several factors, such as part of the plant from which the EO was extracted and season of cultivation, can alter the composition of an EO and the concentrations of each constituent [9–38]. Eugenol and cinnamaldehyde have been reported as the main constituents in EOs of *Cinnamomum* spp. [30, 39, 40]. Accordingly, our analysis by GC–MS of the *C. verum* EO used in our experiments also showed *(E)-*cinnamaldehyde (81.52%) and eugenol (16.68%) as main constituents.

Based on differences in the composition of the EO extracted from leaves of *Cinnamomum osmophloeum*, Cheng et al. (2006) [30] classified it into six chemotypes, according to the main component. The cinnamaldehyde and cinnamaldehyde/cinnamyl acetate types had the strongest antifungal activity, because of a higher concentration of cinnamaldehyde. Singh and colleagues (2007) [40] also attributed the antifungal effect of *Cinnamomum zeylanicum* EO to the high concentration of cinnamaldehyde. The *C. verum* EO that we investigated had mostly cinnamaldehyde in its composition, which suggests that its activity could be related to this component. However, *(E)-*cinnamaldehyde was not effective against *T. cruzi* epimastigotes at low concentrations up to 300 μM (39.648 μg/ml). *(E)-*cinnamaldehyde was active against *T. brucei* trypomastigotes (IC_50_ = 2.93 μg/ml [41]), which indicates different susceptibilities among different pathogenic trypanosomatids and different developmental forms.

The tetrazolium dye MTT can be used to measure cytotoxicity (loss of viable cells: trypanocidal) or cytostatic activity (trypanostatic) of potential drugs. MTT reduction occurs via NAD(P)H-dependent oxidoreductase enzymes located largely in the cytosolic compartment of the cell [42, 43]. MTT reduction is associated not only with mitochondria, but also with the cytoplasm and with non-mitochondrial membranes including the endosome/lysosome compartment and the plasma membrane [42]. Thus, low optical density of MTT staining can indicate low metabolic activity (trypanostatic) or low number of cells (trypanocidal). In our experiments, all plates were observed in inverted microscope before the MTT assay, to assess possible EO activity. In these observations we could see fewer cells and increased cellular debris (as compared with the untreated control) with increasing EO concentration (data not shown). Therefore, our data indicate that *C. verum* EO has a trypanocidal effect.

Activity of cinnamon EO could be associated with the lipophilic characteristic of its constituents. As typical lipophilic molecules, they cross the cell membrane and once inside the cells, cinnamaldehyde could interact with a variety of proteins, forming covalent bonds with amino acid residues, inactivating enzymes, and affecting a number of cellular activities. The mode of action against *T. cruzi* could be via addition of an aldehyde thiol to sulfur-containing components in the key enzymes trypanothione and trypanothione reductase [41], which would lead to a redox imbalance (detected by the MTT assay). Therefore, it is possible that *C. verum* EO acts inside trypanosomes by promoting redox imbalance in the cytosol.

## Conclusions

Biological activity of eight different essential oils was screened against *Trypanosoma cruzi* epimastigotes. The essential oils of *C. verum*, *M. frondosus*, and *E. uniflora* showed the best activity and are promising agents that deserve further study. *C. verum* essential oil was effective on the three developmental forms of *T. cruzi* (epimastigotes, trypomastigotes, and amastigotes) and on the in vitro differentiation of this parasite. *C. verum* essential oil was as effective as, or more effective than, other essential oils or their main constituents tested on trypanosomatids. Evaluation of cinnamaldehyde derivatives is as a potential strategy for further studies to find increased selectivity on *T. cruzi* and identification of the mode of action against this parasite.
